# Virtual Fence System Based on IoT Paradigm to Prevent Occupational Accidents in the Construction Sector

**DOI:** 10.3390/ijerph18136839

**Published:** 2021-06-25

**Authors:** María del Carmen Rey-Merchán, Jesús M. Gómez-de-Gabriel, Antonio López-Arquillos, Juan A. Fernández-Madrigal

**Affiliations:** 1 PhD Program Advanced Computing, Energy and Plasmas, University of Córdoba, 14071 Córdoba, Spain; ep2remem@uco.es; 2 System Engineering and Automation Department, University of Málaga, 29071 Málaga, Spain; jesus.gomez@uma.es (J.M.G.-d.-G.); jafernandez@uma.es (J.A.F.-M.); 3 Economics and Business Management Department, University of Málaga, 29071 Málaga, Spain

**Keywords:** IoT, BLE, beacons, accident, safety, worker, location, virtual fence

## Abstract

Many occupational accidents in construction sites are caused by the intrusion of a worker into a hazardous area. Technological solutions based on RFID, BIM, or UWB can reduce accidents, but they still have some limitations.The aim of the current paper is to design and evaluate a new system of “virtual fences” based on Bluetooth Low-Energy (BLE) to avoid intrusions. First of all, the system was designed using a number of beacons, a Bayesian filter, a finite state machine, and an indicator. Secondly, its safety attributes were evaluated based on a scientific questionnaire by an expert panel following the staticized groups’ methodology. Results showed that the proposal is inexpensive and easy to integrate and configure. The selected experts evaluated positively all the attributes of the system, and provided valuable insights for further improvements. From the experts’ discussions, we concluded that successful adoption of this “virtual fence” system based on BLE beacons should consider the influence of factors such as cost savings, top management support, social acceptance, and compatibility and integration with existing systems, procedures, and company culture. In addition, legislation updates according to technical advances would help with successful adoption of any new safety system.

## 1. Introduction

Occupational accidents are a very important concern at many countries [1]. Several causes of accidents are identified in the literature, such as shortcomings with equipment, deficiencies with risk management, or problems with conditions of materials [2,3,4,5,6]. In order to reduce the negative accidents rates in the sector [7], different preventive strategies have been proposed: safety training [8], collective measures [9], personal protection equipment [10], safety signs [11], etc. However, the ones currently applied in practice are not always enough to prevent all accidents. Although the construction sector is slow in adapting to new technologies in comparison with others, such as manufacturing [12], the higher rates of information and the implementation of advanced communication technologies are expected to improve this trend [13]. In this sense, existing barriers in the sector, such as lack of information, limited technology useful life, or limited attributes and features, are possible to be reduced [14].

Many occupational accidents in construction sites are caused by the accidental intrusion of a worker in a risky zone [15]. Intrusion, defined as unauthorized stepping-into a hazardous area, is considered the prime cause of incidents such as fall from heights or being struck by moving objects [16].

The problem of tracking and location of objects and people indoors has been addressed by several authors [17,18,19]. In order to compare the emerging approaches of the Indoor Positioning system, some authors developed a modeling technique for measuring and comparing effectiveness of cited systems [20]. Other authors reviewed existing real-time location systems, and they found that the majority of them determine the indoor location of a wearable tag via the known location of reference nodes [21]. Other researchers proposed the combination of smartphones with radio beacons and maps to positioning pedestrian in indoor environments [22]. In the particular case of hospitals, their specific solutions are named as Hospital Real-Time Location systems (HRTLS) and are based on different technologies [23]. Similarly, the construction sector has developed some specific solutions adapted to construction sites [24]. For instance, an RTLS based on BLE and accelerometer was designed to improve safety for workers in confined construction sites [25]. Aligned with that, other RTLS are based on other technologies such as Ultra Wide Band [26], RFID [27], or BIM [28].

Nowadays, existing technologies are very useful to detect the presence of a worker in an unsafe zone. It is important to consider the privacy of workers when they are tracked or located. A potential threat to their privacy can reduce its adoption and appropriate use [29]. In accordance, some authors used the concept perceived privacy risk (PR) in their research [30,31]. With regard to technologies, RFID has been used to detect the proximity of the worker to dangerous heavy equipment [32], or the worker stepping into the risk zone by using a detection arch [33], but, when the entrance is wider, it may not be suitable to be covered by such an arch—for example, in a road maintenance operation or a railway construction—preventing the implementation of RFID solutions.

Sensorizing workers by using wearables has been revealed as an alternative and effective strategy to monitor the position of the person [34]. One of the technologies to detect those positions in construction sites is GPS [35]. However, although it can be very effective in open environments, it has two important issues: indoor signals are distorted, and the error range is around meters, while the difference between a safe and an unsafe zone may be of only a few centimeters [36].

Other technologies that involve the use of BIM [37] or UWB [15] are very complex to set-up, and have poor adaptation to changes of the working place, an aspect especially important in construction sites. Then, advantages and disadvantages were identified at every available technologies as it is showed in Table 1.

In the literature, a substantial amount of related works were based on BLE beacons and RFID. RFID combined with wireless sensor networks (ZigBee and 6LowPan protocols) presented some important challenges as their reliability, scalability, and energy consumption [41]. In contrast, the main disadvantage of BLE beacons can be addressed increasing the number of beacons and the sample size at a relatively low cost [17].

A recent approach to the problem is the use of devices to locate workers that periodically broadcast messages, using Bluetooth Low Energy (BLE) radio, commonly named as beacons. Beacons have been successful in particular to monitor the proper use of harnesses by workers in construction sites [38]. In contrast with GPS, the accuracy of BLE beacons is relatively high, and their effectiveness can be proven indistinctly in indoor and outdoor environments. Moreover, their configuration is easier and their installation cheaper, and they are much more suitable for dynamic environments [39].

The aim of this paper is to design and evaluate a new safety system for the detection of workers in risky zones based on BLE beacons as core components. It consists of the creation of a “virtual fence” using these devices. The delimitation of risk zones can be achieved by simply using the relative distance between beacons that can be easily placed in the site. Once the “virtual fence” is defined, the signals processed from the beacons will warn the worker if someone is trespassing.

In the paper, we describe this system and evaluate its feasibility through the answers to a questionnaire by a number of selected experts. We analyze both the questionnaire items and the free discussion conducted with these experts in order to provide a clear and complete account of the possibilities and improvements that our proposal would provide in preventing accidents in construction sites.

This paper is organized as follows: Section 2 describes the design and development of the proposed system, and the methodology to evaluate it based on an expert panel. Section 3 described results from the evaluation carried out by the expert panel, and the discussion of their results. Finally, the main conclusions are highlighted in Section 4.

## 2. Materials and Methods

**Figure 1:**
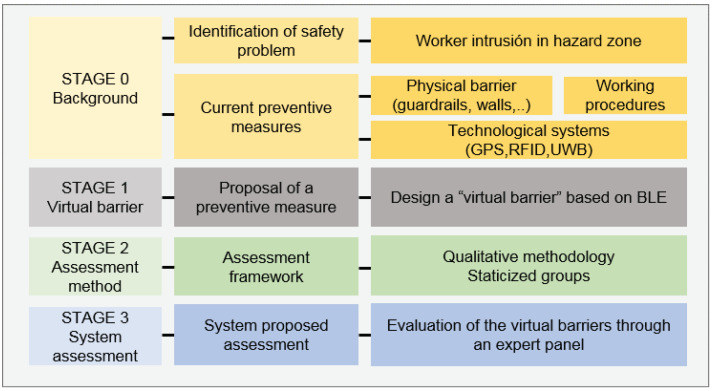
Methodological summary.

Our research methodology has been divided into four main stages, as shown in Figure 1.

- STAGE 0—Background. The safety problem in construction sites consisting of the intrusion into a hazardous zone has been identified, as explained in the Introduction section. Existing preventive measures and relevant previous works have been catalogued (see Section 2.1).
- STAGE 1—Virtual fence system. In this step, a virtual fence system has been devised based on BLE beacons technology. The system is composed of beacons attached to signing cones, a beacon detector carried by the worker as a wearable, and a finite state machine with localization filters implemented in the monitoring software. This is described in Section 2.1.
- STAGE 2—Qualitative assessment design. Once the system has been established, a framework for evaluating it has been designed following the existing literature. The qualitative methodology of choice has been the one of *staticized groups*, i.e., a group of qualified experts to form an assessment panel.
- STAGE 3—Qualitative assessment run. Interviews have been conducted with the experts selected at stage 2. The results have been analyzed and discussed.

In the rest of this section, we describe stages 1 and 2 in more detail. Stage 3 is dealt with in the Results section.

### 2.1. Virtual Fence System Design

Currently, preventive measures to avoid intrusions into risk zones can be classified into three categories: physical barriers (walls, gates, safety rails), safety procedures, and technological location systems (GPS, RFID, UWB, etc). Physical barriers are sometimes difficult to assemble and disassemble in dynamic environments with daily changes, and they may fail [42]. Safety procedures, on the other hand, can be poorly designed or insensitive, and frequently they are not updated according to the changing environments of construction sites [43,44].

Virtual barriers have been implemented using different methods, such as infrared light beams to limit the working area of automatic indoor cleaning robots and global positioning methods (i.e., GPS), which are suitable for outdoor environments but with only moderate accuracy, and dual beacon arrangements with isolation metal plates [45].

Local methods that only provide relative positioning have some advantages over global ones in terms of flexibility, ease of deployment, and privacy. They can be implemented with passive (i.e., receiver only) wearables, and notify the user without providing ID or location information to the system [46].

Although particular localization technologies have their own disadvantages—RFID have low precision and GPS are only recommended for outdoors [47]—BLE has interesting features as well: low cost, good maintainability, stability, good accuracy, and low power usage. Therefore, this has been our choice for the virtual fence proposal.

**Figure 2:**
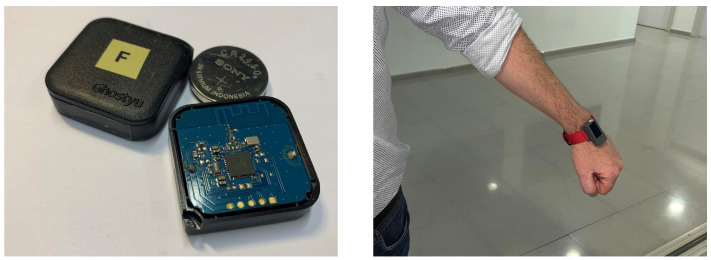
BLE Beacon (**left**) and an ESP32-based wearable receiver used to implement the virtual fence detection (**right**).

**Figure 3:**
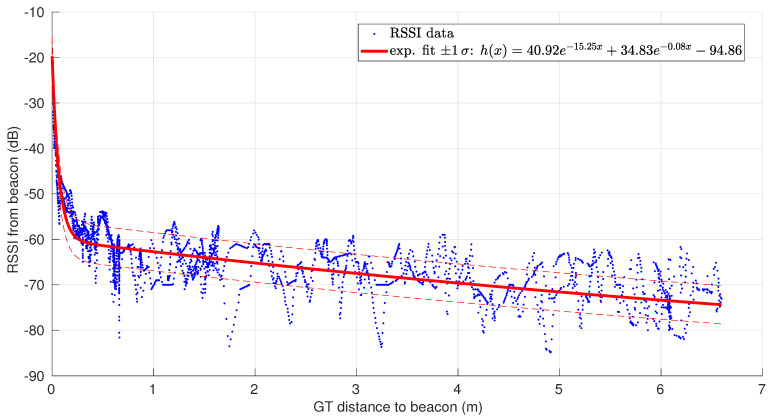
Readings of energy level from a BLE beacon vs. ground-truth distances (provided by LIDAR) in a scenario where we moved away from the beacon and then came back. An exponential sensor model is also shown [38].

An example of BLE beacon and wearable receiver is shown in Figure 2. It is possible to retrieve the distance of the wearable to the beacon from energy level readings by a suitable exponential model, as depicted in Figure 3.

The new system proposed is based on an IoT paradigm. The IoT concept can be defined as a “network of items each embedded with sensors which are connected to the Internet” [48]. The most extended architectural model of IoT systems is based on a three tier framework: sensing, network, and application [49]. The main elements included in the proposed system are sensors (BLE), receiver, IoT broker (MQTT), WI-FI network, a Bayesian filter, and a Finite state machine. From the application layer, relevant information for safety management will be obtained. For instance, if the worker crossed the virtual fence from a safety zone to a dangerous one, the system will detect it, and it will warn the worker. This information can reduce fatal accidents linked to a fall from heights. In addition, exposure time to the worker near of the delimited zone by the virtual fence will be tracked too.

**Figure 4:**
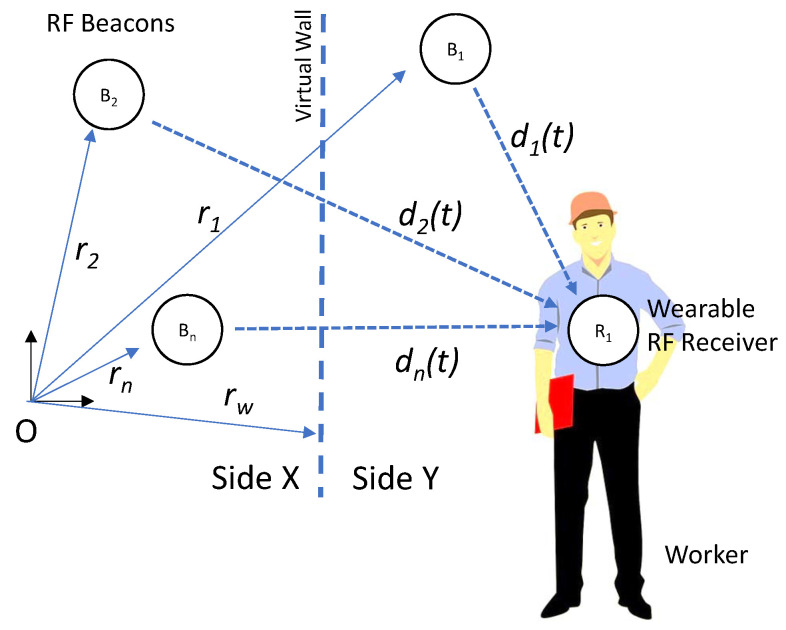
Schematic of the virtual fence system.

For designing our virtual fence system, it has been necessary to choose the number of beacons, the reception technology, data transmission and recording, and also to define a finite state machine and localization methods to be implemented in order to locate the worker in a robust manner. For the proposal in this paper, we rely on technical research done previously on the detection of entering risky zones in construction sites through the use of isolated (non-virtual fence based) BLE beacons [38]. Figure 4 shows a general scheme of the “virtual fence” system that can be constructed upon those results.

The proposed virtual fence implementation is based on a set of RF beacons $B_{i}$ with fixed position relative to the virtual barrier, as shown in Figure 4 and described in Table 2; the user is equipped with a receiver $R_{1}$ (e.g., wearable RF receiver) and an embedded microcontroller unit (MCU), which estimates the relative distances $d_{i}\left(t\right)$ to the beacons. The chosen MCU is an ESP32 Chip-set (Espressif Systems, Ltd, Shanghai, China) integrated into a commercial wearable development system (M5Stick from M5Stack, Guangdong, China). This small device features a 240 MHz dual-core, 600 DMIPS processor with WI-FI/Bluetooth communications. It also includes an 80 mAh LiPo battery, display, and an Inertial Measurement Unit (IMU). It can be programmed under an Open Source programming platform (Arduino, from Arduino.cc), taking advantage of the large availability of libraries and programming resources.

These distances can the used to determine the position of the receiver relative to the Virtual Barrier.

**Figure 5:**
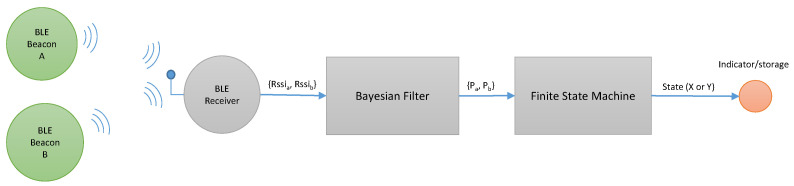
An example of a virtual fence formed by placing two BLE beacons in sequence and using a BLE wearable receiver to notify the user when a change of area occurs.

For increased robustness in the detection of the worker passing near the beacons, the MCU system implements a Bayesian filter for the distance estimation to each beacon plus a finite state machine (FSM) for the detection of state changes in those distances (as shown in Figure 5 and was demonstrated technically in [38]). The results of the localization process can be notified about the user using, for instance, an acoustic indicator.

The Bayesian filter is the union of two different filters—an approximation to the true estimation problem for reducing computational cost: firstly, an EKF is in charge of estimating $d_{k}$, the metrical distance from the receiver to the beacon at step *k*; then, a discrete (also Bayesian and recursive) filter designed for this problem uses the result of the first filter as an observation in order to estimate the distribution of the binary variable $c_{k}$ that models whether the receiver and the beacon are close or far from each other.

The implementation of the EKF is the standard one that can be found in any textbook (see, e.g., [50]); thus, at each step *k*, a prediction on the beacon–receiver distance is made that is updated by a correction stage. For this, we need to provide concrete values for $Q_{k}$ (motion uncertainty), the Jacobian of $h_{k}$ (observation model), and $R_{k}$ (sensor uncertainty). The two latter ones are just the derivative of and the variance of $h_{k}\left(x\right)$ when *x* is instantiated with a particular value of beacon–receiver distance, respectively, both already defined in our observation model shown in Figure 3. Regarding $Q_{k}$, under the *non-motion model* of transition, it is known that $Q_{k}={\hat{v}}_{max}^{2}/\chi_{1,\alpha }^{2}$ if the expected maximum speed of the system (the worker in this case) is ${\hat{v}}_{max}$ with a probability of 1−α. In our case, we have chosen 0.5 m/s as the maximum worker speed at all times with 95% of probability (α=0.05), thus we obtain Q=0.067.

As for the discrete filter, the equation to implement is as follows, where $z_{k}$ comes from the EKF at the same step (it is the probability of the distance beacon–receiver $d_{k}$ to be smaller than a threshold $\tau_{c}$ that we set at 70 cm, i.e., $z_{k}=cdf_{EKF_{k}}\left(\tau_{c}\right)$):

(1) $$\underset{\mathrm{posterior}\;\mathrm{at}\;k}{\underset{︸}{P(c_{k}|z_{1:k},d_{1:k})}}\;\propto \;\underset{likelihood}{\underset{︸}{p(z_{k}|c_{k})}}\;\underset{prior}{\underset{︸}{\;\sum_{k-1}\;\underset{transition}{\underset{︸}{P(c_{k}|c_{k-1},d_{k})}}\;\underset{\mathrm{posterior}\;\mathrm{at}\;k-1}{\underset{︸}{P(c_{k-1}|z_{1:k-1},d_{1:k-1})}}}}$$

Here, we define these probabilities and parameters:

- $p(z_{k}|c_{k})$ is the likelihood of being closer than the threshold provided that we know we are close or far. The shape of this likelihood and its parameters are designed as a first-order system step response, as explained in detail in [38].
- $P(c_{k}|c_{k-1},d_{k})$ is the probability of changing from close to, far, or between any other combination of close/far in one step of motion, provided that we know the metrical distance between beacon and receiver. We approximate this (since the value of $d_{k}$ is unknown) as a modulation of the simpler $P(c_{k}|c_{k-1}$ through $d_{k}$: $P(c_{k}|c_{k-1})$ is increased linearly if the evidence provided by the estimate of $d_{k}$ supports the particular combination of $c_{k-1}$ and $c_{k}$, and is decreased linearly when that evidence contradicts it (again, see details in [38]).
- The prior distributions for $d_{0}$ and $c_{0}$ are not critical, since the filters usually converge in a few steps. In our experiments, we have chosen values compatible with practical scenarios, in particular for the worker being initially around 10 cm from the true distance from the beacons with 95% probability and, consequently, in state $c_{0}=\mathrm{close}$ with 100% probability.

**Figure 6:**
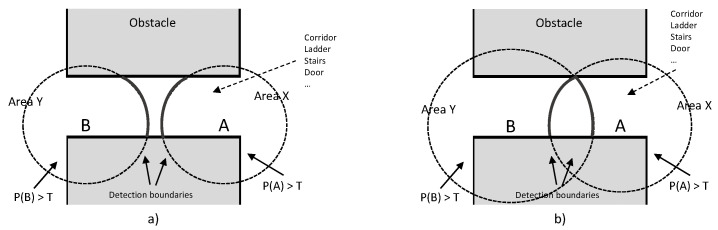
Beacons A and B define a virtual barrier in the transition between a safe place and a risk area. Proximity areas are defined by the proximity function. In (**a**), the proximity areas to A and B are separate. In (**b**), the two proximity areas overlap [38].

The virtual fence can be formed by placing beacons along a corridor or gate that links safe and risk areas. In Figure 6a, particular geometrical arrangement of the beacons *A* and *B* along a corridor is shown, with different distances related to the received power (RSSI) P(A) and P(B) and the proximity threshold *T*. In the figure, we both consider non-overlapped and overlapped areas (Figure 6a,b, respectively).

**Figure 7:**
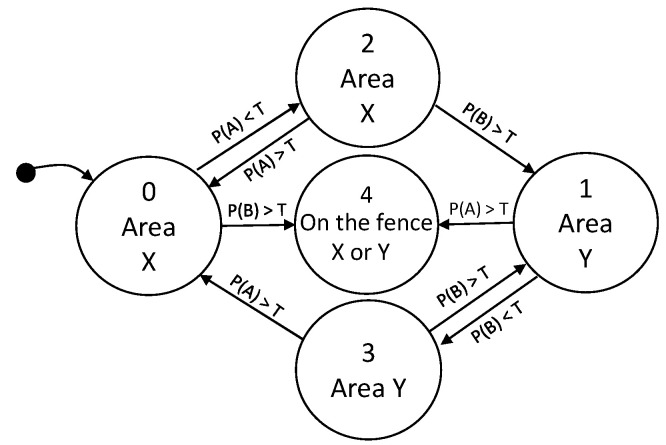
Finite state machine that estimates the location of a receiver relative to the virtual fence of Figure 6. Transitions are based on the proximity function to beacons A and B [38].

The Finite State Machine that complements and robustifies the relative distance estimations, based on the threshold *T*, from the worker to the beacons made by the Bayesian filter is shown, as a simplified diagram, in Figure 7. With this approach, the actual side of the user can be deduced even when the user crosses and then gets far from the barrier, and can even take into account the fact that the path should be travelled again in reverse order afterwards. In this way, the risk exposure time and other measures of risk assessment can be properly and consistently recorded.

#### Scenarios for Potential Application in the Construction Sector

**Figure 8:**
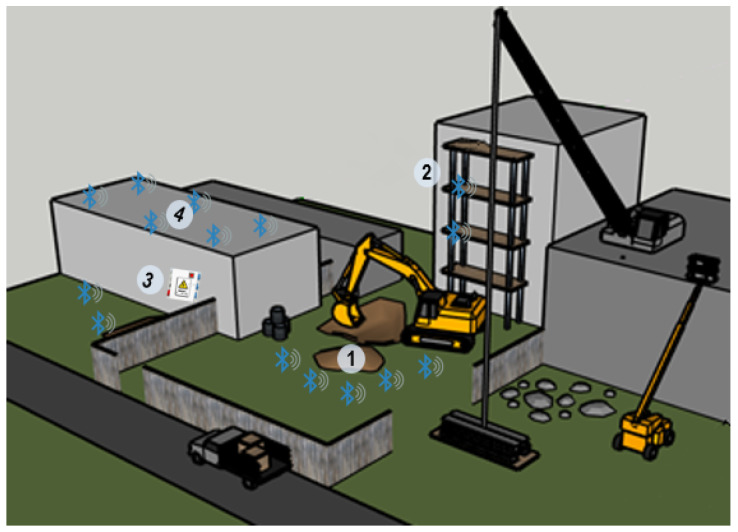
Usage scenarios in a construction site.

The system proposed presented an easy configuration in different potential construction scenarios. Then, its possible applicability against safety risks is not limited to only a very restricted circumstances. Some of these potential scenarios were sketched in Figure 8 and described in the following figures.

**Figure 9:**
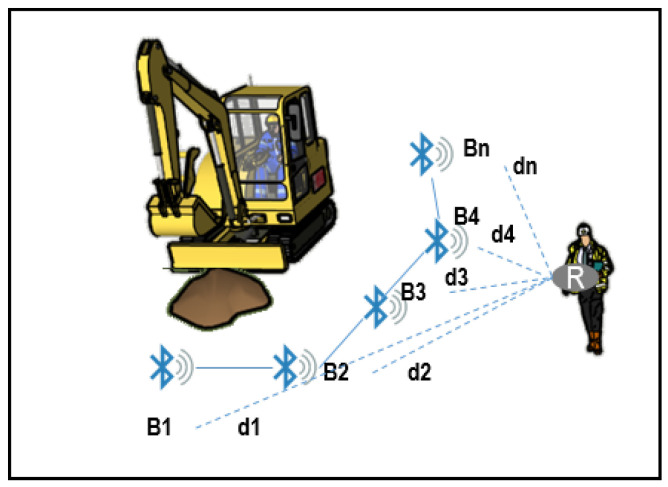
Scenario 1. Virtual fence on excavation works.

In scenario 1 (Figure 9), the virtual fence system was configured to avoid the risk of falling and being struck by linking to an accidental intrusion in the dangerous zone close to the excavation and the heavy equipment. Worker was wearing a receiver (R), and the dangerous zone was delimited by five beacons, although the fence could be extended until the number of beacons needed (n). Distance from workers to the beacons was calculated by receiver (R) attached to the worker and processed by the system.

**Figure 10:**
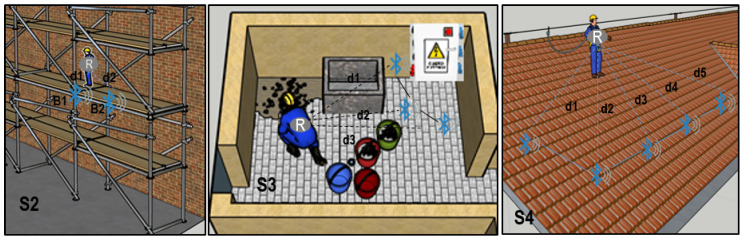
Different usage scenarios of the virtual fence on scaffolding task (S2), with electric risk (S3) and roofing (S4).

In the second usage scenario (S2) (Figure 10), the system was placed in a scaffold structure. Virtual fence was set in the edge of the platform, and the receiver was attached to the worker. Similarly, in the third scenario (S3), electric risks were delimited by beacons. Finally, the system was suitable to improve roofing tasks, as it was described in scenario four (S4).

### 2.2. Qualitative Assessment of the Proposal

The effectiveness of the system proposed in Section 2.1 has been evaluated following the methodology by Hallowell and Gambatese [51]. The *staticized groups* method is a systematic research technique for obtaining the judgement of a panel of independent experts on a topic. These experts are selected according to predefined requirements, and asked to participate in a structured survey in only one round—if the number of rounds is two or more, it is known as the *Delphi method*.

Staticized groups improve simple surveys because respondents are certified previously as experts. The method is recommended when objective data are not possible to obtain (as it happens in the current state of development of our proposal), or experimental tests are not realistic or ethical (as it happens in reality when a worker is in a risky situation). In particular, it has been identified as a suitable methodology for validating results of a safety intervention without exposing construction workers to increased safety risks [51]. The procedure was summarized by Hallowell and Gambatese [51]. This is to be instantiated to evaluate the applicability and effectiveness of the previously described system of virtual fences for safety in construction works, as we explain in the following.

#### 2.2.1. Panel Members Selection

A crucial step is to identify potential experts for the evaluation of our approach. Their level of expertise is a very important trait; thus, a flexible scoring system, based on the expert achievements and experience in certain categories, has been used for their scoring (see Table 3).

Following the guidelines proposed by Hallowell and Gambatese [51], the panelists should score at least 1 in four different categories, and a minimum of 11 total points in order to be selected as valid. The number of panelists suggested in the literature [52] should be at least 8 and not more than 16. During the selection process, some experts may not complete the round.

According to this procedure, the authors have contacted 14 construction business and three universities. Once the background and availability of the candidates have been reviewed, 12 experts have been selected according to the guidelines previously described. Three experts from among them did not pass the procedure in the end. The general qualifications of the panelists are as follows:

- All have a Master’s degree in Occupational Health and Safety, which demonstrates their training in Safety topics.
- All have a Degree or a Master’s with a technical profile.
- All panelists together have a total experience of 139 years.
- Three of them have published more than five scientific papers related with construction safety.

In Table 4, you can see the detailed scoring of every expert.

#### 2.2.2. Questionnaire Design

The questionnaire that the experts selected in Section 2.2.1 have to answer has been designed by including the main aspects of an existing one that assesses *poka-yoke* devices, proposed by Saurin [53]. Poka-yoke can be defined as a system to easily avoid failure and mistakes in the workplace [54]. According to literature guidelines [55], an equipment with a warning system, such as the virtual fences proposed in this paper, can be considered as a poka-yoke device.

This approach provides a scoring system to perform the assessment rigorously, based on a number of *attributes* with several possible answers that score differently. In addition to the questionnaire, all of our experts have been invited to explain and justify their answers, and we have added their comments about any aspect they considered relevant.

A rigorous research should control the bias in the answers to the previous questionnaire. In order to minimize the effects of these biases, we have used the controls summarized in Table 5.

## 3. Results and Expert Discussion

The questionnaire results obtained from the experts are summarized in Table 6. In the following, we detail and comment on them. In order to test the reliability of the questionnaire, Cronbach’s Alpha was calculated (0.777). This value can be considered as adequate or high [56]. Cronbach’s alpha if the item is deleted and correlations are shown in Table 7.

The average obtained from the attributes’ scores and showed in the last row of Table 5 are very close to those obtained from the other safety system assessed previously [53] using a similar questionnaire. In the cited research, a presence sensor installed in a press obtained 3.12 points, and the access gate to the freight elevator scored 2.94 [53].

In our case, all experts noticed that the system only has the warning function. Warning functions such as intrusion alert technologies can improve worker safety [57], but their effectiveness can be reduced by some factors, e.g., high levels of noise or frequent false alarm. Some interviewed experts pointed out that the effectiveness of the alarm is linked to the perception of its usefulness by the worker. One of them said: *“Workers with low risk perception probably will ignore the alarm”*. Similarly, another professional added: *“If the worker is not properly trained about safety risks, he will not react properly to the warning signal”*.

However, our proposal includes a functionality which could be considered as a control function (although it is evident that respondents did not appreciate that as being very useful). Particularly, the system can record when and how many times a worker enters a dangerous zone, and these data can help the foreman with planning future tasks. The experts considered that this input does not allow for controlling worker actions in real time, and this is the reason that they did not consider it as an effective control tool. It is interesting that, being asked about this issue, one of them commented regarding the limited practical utility of *any* control system: *“It is not possible to control a worker as a machine; you can not turn him off like a device, you can only warn him about a risk. He makes the last decision.”*. Control strategies are obviously more useful on machines because you can modify parameters such as its speed [58,59], but we claim that, since unsafe worker behavior is the major cause of fatalities in construction accidents [60], the improvement of *supervisory control* factors such as project management, or task planning and scheduling, may effectively contribute to reducing the likelihood of accidents [61]. According to that, although total control over worker behavior is not possible, the system proposed here supports and improves this kind of supervision, and therefore it would enhance worker safety through an indirect modification of behaviors.

All interviewed experts agreed that the system positively impacts the workers, no one considering that the system does not improve their safety conditions. However, some doubts were exposed about the adoption of the system by companies and workers if it *only* improves safety. *“Companies are more likely to invest in technology when they identify cost saving. Only potential safety benefits many times is not enough”*. This opinion matches with previous studies that identified cost saving potential as a primary factor to adopt technology in the sector [29]. In spite of previous research demonstrating that, for every £1 spent on accident prevention, contractors obtain £3 as benefit [62], some authors still find that the cost of accidents by themselves might not be enough to influence firms to invest in safety prevention [3].

Another primary factor mentioned by our experts in this regard has been the influence of managerial support *“The final success or failure of the system adoption will be affected by top management support. If they do not trust in the system, it will be disappear sooner than later. Positive impact on workers is not the overriding factor”*. This opinion is in concordance with studies that indicate the top management support is an important factor to adopt technologies in construction [63]. In this sense, some experts extend the positive impact of the proposed system to the rest of the staff as well, not only to the workers with a receptor attached. *“Safety attitudes generate higher safety perception”* was mentioned. This can be motivated because of the influence of intention and social norms in worker behaviors [64]. As a preventive measure, the system proposed here can be classified as Personal Protection Equipment: if a beacon receptor is supplied to every worker, the virtual fence will have the same effect as a collective measure.

On another level, one expert commented: *“Some workers could have the perception that the beacon is a way to monitor their location and tasks performance in order to control their productivity more than as a safety tool. It is important an explanation of the tool to avoid misunderstandings."*. Workers’ privacy is a key factor in a monitoring device. It was found that a potential threat to their privacy can reduce its adoption [29]. Indeed, social acceptance has been revealed as a challenge in existing wearable systems [65]. A proper use of data ensuring anonymity will help to improve the social acceptance of our proposal. Aligned with that perspective, it has been pointed out elsewhere that the key to technology adoption is the integration in the processes of the organization, systems, and cultures rather than technology itself [66].

With regard to the performance of the system, a majority of respondents considered that a specific action of the worker for its proper use is necessary, for example to attach the beacon as a wearable and turn it on. Only two experts considered that any action to activate the system was not necessary. Asked about this opinion, they explained that, if the activation of the system is done at the beginning of the workday, this step can be considered prior to its normal use during the day.

A total consensus has been detected in the results concerning the safety risks for the worker. Everybody agrees that the system does not introduce any additional risks to the worker.

In terms of maintenance, a majority of experts have considered that the calibration or replacement should be included in maintenance plans as it occurs in comparable systems [53].

In addition to the questions included in the survey of Section 2.2.2, some of the experts pointed out that the effectiveness of the system will be conditioned by particular barriers existing in the sector. Concretely, construction sites are dynamic production environments, can employ people with poor training, and there is the general industry resistance to change, as pointed out by an expert: *“Technology adoption culture in a dynamic construction site can not be compared with technological culture in the manufacturing sector. Firms’ usual procedures and workers set of minds are very different”*. This fact is not new; it has been argued previously that some aspects of the integration of technologies process in construction are different from other sectors [67].

One of the respondents said that *“Although the tool could be effective, employee privacy concern could be a problem; it would be necessary a previous agree with Unions”*. This concern about privacy and electronic gadgets at work is aligned with other authors as well [68], and it is related to the previously issues mentioned about the worker perceptions on their privacy.

Another panelist showed his special concern about legal regulations: *“If the legislation establishes that physical barriers are compulsory to prevent some specific risks, construction companies probably will not spend extra money in additional preventive measures, unless they obtained some additional profits using the system such as safety coordination or productivity.”*. The quoted concern about safety regulations in the sector is not new either; the problem is extended and frequently related to electronic safety applications, as it occurs with virtual reality or augmented reality [69]. In many cases, legislation is outdated in relation to technical advances. Therefore, legislation updates according to the current technological advances would be necessary.

## 4. Conclusions

A virtual fence system based on BLE beacons was designed and evaluated by an expert panel. It was composed of a set of RF beacons with a fixed position, a receiver attached to the worker, and an embedded MCU that estimates the distance to the beacons. For a higher robustness, a Bayesian Filter for the distance estimation and a finite state machine were added to the system.

The proposal was considered suitable for different scenarios in construction projects such as excavation, scaffolding, and roofing. The system we propose could improve safety in construction sites by delimiting risk zones and detecting and warning about worker intrusions in a robust manner. The system is cheap, lightweight to be integrated in worker equipment, easy to configure, and it does not interfere with production tasks. Its maintenance does not require special effort, and it can be adapted to the dynamic scenarios of most construction sites.

The experts selected have evaluated positively all attributes of the system, and provided valuable insights into its attributes. The barriers they have found in the acceptance of the system are very similar to the ones of other comparable technologies, such as RFID, Virtual Reality, or Augmented reality.

A successful adoption of the proposed virtual fence system should consider the identification of crucial factors provided by the experts, such as cost savings. The savings of avoiding an accident, linked to the costs of the coordination and planning involved in the solution, seems to produce a good balance to adopt it. Top management support is another important factor considered by some experts, which would require clear explanations of the utility and ease of use and configuration of the system. Social acceptance is identified as a challenge whenever a new procedure or technology is proposed. Thus, it is important that all stakeholders (workers, foremen, managers, safety coordinators, etc.) perceive the system as a useful tool, knowing their advantages and potential benefits. Data obtained must be computed carefully for respecting privacy in order to obtain a better acceptance of the system. In addition, the compatibility and integration with existing systems, procedures, and company culture will clearly affect the adoption, integration, and effectiveness of this beacon based system.

Last but not least, the resistance to change traditional procedures and the slow technology adaptation in the construction sector can only be effectively overcome through updating the legislation and promoting new techniques from public institutions.

### Future Research

Our current study has unravelled some improvement opportunities, as revealed by the experts.

Although safety systems are frequently difficult to test in construction sites due to ethics and the physical nature of the problems to test (fall from heights), in this case, and under controlled conditions with physical barriers added to the virtual fence, the system could effectively be tested. Implementing and evaluating a prototype in a real construction site could help to develop its functionalities and to amend possible weaknesses.

Additionally, evaluating the social acceptance level between stakeholders will also help, not only in this proposal but in improving existing strategies to extend the use of technological systems in the sector.

Other future research lines also include the integration of the system with other technological options, such as BIM or GPS, with the aim to obtain the best for each one while dealing with their respective lacks.

## Figures and Tables

**Table 1:** Comparison of location technological solutions.

Technology	Advantages	Disadvantages
BLE [18,25,38,39]	Low power, easy to configure, low price, maintainability	Low precision
RFID [27,33]	No battery in tag, low price	reliability, scalability
GPS [35,36]	High coverage, not additional communication support required	Low precision, Only outdoors
BIM [28,37]	Information management	High cost, Complex configuration
UWB [19]	High precision	Complex configuration
COMPUTER VISION [40]	Good accuracy, data acquisition	Workers privacy, Controlled environment only, Human error

**Table 2:** Virtual fence system description.

Parts of System	Function	Units
RF Beacons (Bn)	Beaconing a signal from the virtual fence	RSSI
Wearable RF receiver (R1)	Measurement of signal strength	RSSI
Bayesian filter	Proximity detection	RSSI and distance
Finite state machine	Status detection	Worker status

**Table 3:** Scoring categories for the qualification of experts.

Achievement or Experience	Abbreviation	Score
Years of professional experience	Exp	1 (each year)
Professional registration	Reg	3
Safety manager	Saf	3
Faculty member at university	Uni	3
Bachelor of Science	BS	4
Master of Science	MS	2
Doctor of Science	Ph.D	4

**Table 4:** Summary of experts scoring using Table 3.

Expert	Exp	Reg	Saf	Uni	BS	MS	Ph.D	Total
1	38	3	3	0	4	4	0	52
2	17	3	3	0	4	4	0	31
3	8	3	0	3	4	4	4	26
4	15	3	0	0	4	4	0	26
5	22	3	0	0	4	4	0	33
6	4	0	3	0	4	4	0	15
7	9	3	0	0	4	4	0	20
8	14	0	3	3	4	4	4	22
9	12	3	3	3	4	4	4	33

**Table 5:** Control for bias in our staticized groups procedure.

Control	Affected Bias
Randomize the order of questions for each expert.	Contrast effect and primacy effect.
Remove members who experienced recent events.	Recency effect.
Ensure anonymity of panelist.	Dominance

**Table 6:** Questionnaire results.

Attribute	Average	Median	sd	var
Warning and control function	2.11	2	0.11	0.33
Workers affected	3.11	4	1.05	0.99
Performance	2.44	2	0.88	0.69
Safety risk for workers	4.00	4	0.00	0.00
Maintenance planning	3.78	4	0.67	0.40
Maintenance tests	2.67	2	1.00	0.89
Total system’s attribute	3.02	3	0.66	0.50

sd = standard deviation; var = sample variance.

**Table 7:** Cronbach’s Alpha test results.

Attribute	Correlation	Cronbach’s Alpha if Item Deleted
Warning and control function	0.416	0.777
Workers affected	0.693	0.730
Performance	0.598	0.743
Safety risk for workers	0.449	0.772
Maintenance planning	0.636	0.717
Maintenance tests	0.636	0.717

## Abbreviations

The following abbreviations are used in this manuscript:

BIM	Building Information Management
BLE	Bluetooth Low Energy
GPS	Global Positioning System
IoT	Internet of Things
RFID	Radio Frequency Identification
UWB	Ultra Wide Band

## References

[1] Andersen J.H., Malmros P., Ebbehoej N.E., Flachs E.M., Bengtsen E., Bonde J.P. (2019). Systematic literature review on the effects of occupational safety and health (OSH) interventions at the workplace. Scand. J. Work Environ. Health.

[2] Pietilä J., Räsänen T., Reiman A., Ratilainen H., Helander E. (2018). Characteristics and determinants of recurrent occupational accidents. Saf. Sci..

[3] Forteza F.J., Carretero-Gomez J.M., Sese A. (2017). Occupational risks, accidents on sites and economic performance of construction firms. Saf. Sci..

[4] Haslam R.A., Hide S.A., Gibb A.G., Gyi D.E., Pavitt T., Atkinson S., Duff A.R. (2005). Contributing factors in construction accidents. Appl. Ergon..

[5] Jazayeri E., Dadi G.B. (2017). Construction safety management systems and methods of safety performance measurement: A review. J. Saf. Eng..

[6] Kaassis B., Badri A. (2018). Development of a preliminary model for evaluating occupational health and safety risk management maturity in small and medium-sized enterprises. Safety.

[7] Lombardi M., Fargnoli M., Parise G. (2019). Risk profiling from the european statistics on accidents at work (ESAW) accidents’ databases: A case study in construction sites. Int. J. Environ. Res. Public Health.

[8] Demirkesen S., Arditi D. (2015). Construction safety personnel’s perceptions of safety training practices. Int. J. Proj. Manag..

[9] Ibarrondo-Dávila M., López-Alonso M., Rubio-Gámez M. (2015). Managerial accounting for safety management. The case of a Spanish construction company. Saf. Sci..

[10] Arcury T.A., Summers P., Rushing J., Grzywacz J.G., Mora D.C., Quandt S.A., Lang W., Mills T.H. (2015). Work safety climate, personal protection use, and injuries among Latino residential roofers. Am. J. Ind. Med..

[11] Saurin T.A., Formoso C.T., Cambraia F.B. (2008). An analysis of construction safety best practices from a cognitive systems engineering perspective. Saf. Sci..

[12] Navon R., Sacks R. (2007). Assessing research issues in automated project performance control (APPC). Autom. Constr..

[13] Karakhan A., Xu Y., Nnaji C., Alsaffar O. (2019). Technology Alternatives for Workplace Safety Risk Mitigation in Construction: Exploratory Study. Advances in Informatics and Computing in Civil and Construction Engineering.

[14] Nnaji C., Karakhan A.A. (2020). Technologies for safety and health management in construction: Current use, implementation benefits and limitations, and adoption barriers. J. Build. Eng..

[15] Cheng T., Teizer J. (2013). Real-time resource location data collection and visualization technology for construction safety and activity monitoring applications. Autom. Constr..

[16] Heng L., Shuang D., Skitmore M., Qinghua H., Qin Y. (2016). Intrusion warning and assessment method for site safety enhancement. Saf. Sci..

[17] Huh J.H., Seo K. (2017). An indoor location-based control system using bluetooth beacons for IoT systems. Sensors.

[18] Baronti P., Barsocchi P., Chessa S., Mavilia F., Palumbo F. (2018). Indoor bluetooth low energy dataset for localization, tracking, occupancy, and social interaction. Sensors.

[19] Kolakowski J., Djaja-Josko V., Kolakowski M., Broczek K. (2020). UWB/BLE tracking system for elderly people monitoring. Sensors.

[20] Ang J.L.F., Lee W.K., Ooi B.Y. GreyZone: A Novel Method for Measuring and Comparing Various Indoor Positioning Systems. Proceedings of the 2019 International Conference on Green and Human Information Technology (ICGHIT).

[21] Loveday A., Sherar L.B., Sanders J.P., Sanderson P.W., Esliger D.W. (2015). Technologies that assess the location of physical activity and sedentary behavior: A systematic review. J. Med. Internet Res..

[22] Herrera J.A., Plöger P.G., Hinkenjann A., Maiero J., Flores M., Ramos A. Pedestrian indoor positioning using smartphone multi-sensing, radio beacons, user positions probability map and IndoorOSM floor plan representation. Proceedings of the 2014 International Conference on Indoor Positioning and Indoor Navigation (IPIN).

[23] Gholamhosseini L., Sadoughi F., Safaei A. (2019). Hospital real-time location system (A practical approach in healthcare): A narrative review article. Iran. J. Public Health.

[24] Kim H., Han S. (2018). Accuracy improvement of real-time location tracking for construction workers. Sustainability.

[25] Lim J.S., Song K.I., Lee H.L. (2016). Real-time location tracking of multiple construction laborers. Sensors.

[26] Umer W., Siddiqui M.K. (2020). Use of Ultra Wide Band Real-Time Location System on Construction Jobsites: Feasibility Study and Deployment Alternatives. Int. J. Environ. Res. Public Health.

[27] Valero E., Adán A., Cerrada C. (2015). Evolution of RFID applications in construction: A literature review. Sensors.

[28] Ratajczak J., Riedl M., Matt D.T. (2019). BIM-based and AR application combined with location-based management system for the improvement of the construction performance. Buildings.

[29] Choi B., Hwang S., Lee S. (2017). What drives construction workers’ acceptance of wearable technologies in the workplace?: Indoor localization and wearable health devices for occupational safety and health. Autom. Constr..

[30] Wang X., White L., Chen X., Gao Y., Li H., Luo Y. (2015). An empirical study of wearable technology acceptance in healthcare. Ind. Manag. Data Syst..

[31] Li H., Gupta A., Zhang J., Sarathy R. (2014). Examining the decision to use standalone personal health record systems as a trust-enabled fair social contract. Decis. Support Syst..

[32] Teizer J., Golovina O., Wang D., Pradhanang N. (2015). Automated collection, identification, localization, and analysis of worker-related proximity hazard events in heavy construction equipment operation. Proceedings of the International Symposium on Automation and Robotics in Construction.

[33] Kelm A., Laußat L., Meins-Becker A., Platz D., Khazaee M.J., Costin A.M., Helmus M., Teizer J. (2013). Mobile passive Radio Frequency Identification (RFID) portal for automated and rapid control of Personal Protective Equipment (PPE) on construction sites. Autom. Constr..

[34] Awolusi I., Marks E., Hallowell M. (2018). Wearable technology for personalized construction safety monitoring and trending: Review of applicable devices. Autom. Constr..

[35] Moselhi O., Bardareh H., Zhu Z. (2020). Automated data acquisition in construction with remote sensing technologies. Appl. Sci..

[36] Razavi S.N., Moselhi O. (2012). GPS-less indoor construction location sensing. Autom. Constr..

[37] Martinez-Aires M.D., Lopez-Alonso M., Martinez-Rojas M. (2018). Building information modeling and safety management: A systematic review. Saf. Sci..

[38] Gomez-de Gabriel J.M., Fernández-Madrigal J.A., Lopez-Arquillos A., Rubio-Romero J.C. (2019). Monitoring harness use in construction with BLE beacons. Measurement.

[39] Rey-Merchán M.d.C., Gómez-de Gabriel J.M., Fernández-Madrigal J.A., López-Arquillos A. (2020). Improving the prevention of fall from height on construction sites through the combination of technologies. Int. J. Occup. Saf. Ergon..

[40] Morar A., Moldoveanu A., Mocanu I., Moldoveanu F., Radoi I.E., Asavei V., Gradinaru A., Butean A. (2020). A comprehensive survey of indoor localization methods based on computer vision. Sensors.

[41] Landaluce H., Arjona L., Perallos A., Falcone F., Angulo I., Muralter F. (2020). A review of iot sensing applications and challenges using RFID and wireless sensor networks. Sensors.

[42] Winge S., Albrechtsen E. (2018). Accident types and barrier failures in the construction industry. Saf. Sci..

[43] Dekker S. (2003). Failure to adapt or adaptations that fail: Contrasting models on procedures and safety. Appl. Ergon..

[44] Harvey E.J., Waterson P., Dainty A.R. (2018). Beyond ConCA: Rethinking causality and construction accidents. Appl. Ergon..

[45] White S. (2016). Virtual Barrier System and Method. U.S. Patent.

[46] Kapadia A., Henderson T., Fielding J.J., Kotz D. (2007). Virtual walls: Protecting digital privacy in pervasive environments. Proceedings of the International Conference on Pervasive Computing.

[47] Lin P., Li Q., Fan Q., Gao X. (2013). Real-time monitoring system for workers’ behavior analysis on a large-dam construction site. Int. J. Distrib. Sens. Netw..

[48] Singh A., Payal A., Bharti S. (2019). A walkthrough of the emerging IoT paradigm: Visualizing inside functionalities, key features, and open issues. J. Netw. Comput. Appl..

[49] Logvinov O., Kraemer B., Adams C., Heiles J., Stuebing G., Nielsen M., Mancuso B. (2016). Standard for an Architectural Framework for the Internet of Things (IOT) IEEE p2413.

[50] Fernández-Madrigal J.A. (2012). Simultaneous Localization and Mapping for Mobile Robots: Introduction and Methods: Introduction and Methods.

[51] Hallowell M.R., Gambatese J.A. (2009). Qualitative research: Application of the Delphi method to CEM research. J. Constr. Eng. Manag..

[52] Rowe G., Wright G. (1999). The Delphi technique as a forecasting tool: Issues and analysis. Int. J. Forecast..

[53] Saurin T.A., Ribeiro J.L.D., Vidor G. (2012). A framework for assessing poka-yoke devices. J. Manuf. Syst..

[54] Shingo S. (1986). Zero Quality Control: Source Inspection and the Poka-Yoke System.

[55] Saurin T.A., Formoso C.T., Cambraia F.B. Towards a common language between Lean production and safety management. Proceedings of the IGLC-14.

[56] Taber K.S. (2018). The use of Cronbach’s alpha when developing and reporting research instruments in science education. Res. Sci. Educ..

[57] Nnaji C., Gambatese J., Lee H.W., Zhang F. (2019). Improving construction work zone safety using technology: A systematic review of applicable technologies. J. Traffic Transp. Eng..

[58] Thomas L.J., Srinivasan R., Decina L.E., Staplin L. (2008). Safety effects of automated speed enforcement programs: Critical review of international literature. Transp. Res. Rec..

[59] Soole D.W., Watson B.C., Fleiter J.J. (2013). Effects of average speed enforcement on speed compliance and crashes: A review of the literature. Accid. Anal. Prev..

[60] Zhang P., Li N., Jiang Z., Fang D., Anumba C.J. (2019). An agent-based modeling approach for understanding the effect of worker-management interactions on construction workers’ safety-related behaviors. Autom. Constr..

[61] Khosravi Y., Asilian-Mahabadi H., Hajizadeh E., Hassanzadeh-Rangi N., Bastani H., Behzadan A.H. (2014). Factors influencing unsafe behaviors and accidents on construction sites: A review. Int. J. Occup. Saf. Ergon..

[62] Ikpe E., Hammon F., Oloke D. (2012). Cost-benefit analysis for accident prevention in construction projects. J. Constr. Eng. Manag..

[63] Gambatese J.A., Hallowell M. (2011). Factors that influence the development and diffusion of technical innovations in the construction industry. Constr. Manag. Econ..

[64] Goh Y.M., Ubeynarayana C.U., Wong K.L.X., Guo B.H. (2018). Factors influencing unsafe behaviors: A supervised learning approach. Accid. Anal. Prev..

[65] Korman D.B., Zulps A. (2017). Enhancing Construction Safety Using Wearable Technology. Proceedings of the ASSE Professional Development Conference and Exposition.

[66] Loosemore M. (2014). Improving construction productivity: A subcontractor’s perspective. Eng. Constr. Archit. Manag..

[67] Aouad G., Ozorhon B., Abbott C. (2010). Facilitating innovation in construction: Directions and implications for research and policy. Constr. Innov..

[68] Reid C.R., Schall M.C., Amick R.Z., Schiffman J.M., Lu M.L., Smets M., Moses H.R., Porto R. Wearable Technologies: How Will We Overcome Barriers to Enhance Worker Performance, Health, In addition, Safety?. Proceedings of the Human Factors and Ergonomics Society Annual Meeting.

[69] Li X., Yi W., Chi H.L., Wang X., Chan A.P. (2018). A critical review of virtual and augmented reality (VR/AR) applications in construction safety. Autom. Constr..

